# Evidence of proteins, chromosomes and chemical markers of DNA in exceptionally preserved dinosaur cartilage

**DOI:** 10.1093/nsr/nwz206

**Published:** 2020-01-12

**Authors:** Alida M Bailleul, Wenxia Zheng, John R Horner, Brian K Hall, Casey M Holliday, Mary H Schweitzer

**Affiliations:** 1 Key Laboratory of Vertebrate Evolution and Human Origins, Institute of Vertebrate Paleontology and Paleoanthropology, Chinese Academy of Sciences, Beijing 100044, China; 2 CAS Center for Excellence in Life and Paleoenvironment, Beijing 100044, China; 3 Department of Biological Sciences, North Carolina State University, Raleigh, NC 27695, USA; 4 Honors Program, Chapman University, Orange, CA 92866, USA; 5 Department of Biology, Dalhousie University, Halifax, B3H 4R2, Canada; 6 Department of Pathology and Anatomical Sciences, University of Missouri, Columbia, MO 65211, USA; 7 North Carolina Museum of Natural Sciences, Raleigh, NC 27601, USA; 8 Department of Geology, University of Lund, 22362, Sweden

**Keywords:** cartilage, dinosaur, nuclei, chromosomes, collagen II, DNA markers

## Abstract

A histological ground-section from a duck-billed dinosaur nestling (*Hypacrosaurus stebingeri*) revealed microstructures morphologically consistent with nuclei and chromosomes in cells within calcified cartilage. We hypothesized that this exceptional cellular preservation extended to the molecular level and had molecular features in common with extant avian cartilage. Histochemical and immunological evidence supports *in situ* preservation of extracellular matrix components found in extant cartilage, including glycosaminoglycans and collagen type II. Furthermore, isolated *Hypacrosaurus* chondrocytes react positively with two DNA intercalating stains. Specific DNA staining is only observed inside the isolated cells, suggesting endogenous nuclear material survived fossilization. Our data support the hypothesis that calcified cartilage is preserved at the molecular level in this Mesozoic material, and suggest that remnants of once-living chondrocytes, including their DNA, may preserve for millions of years.

## INTRODUCTION AND HISTOLOGICAL OBSERVATIONS

**Figure 1.**
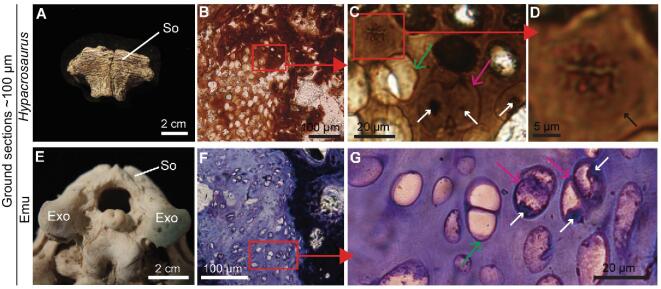
Ground section of *Hypacrosaurus* (MOR 548) supraoccipital shows exceptional histological preservation of calcified cartilage. (A) An isolated supraoccipital (So) of *Hypacrosaurus* in dorsal view. (B–D) Ground section of another So showing calcified cartilage with hypertrophic chondrocyte lacunae. (C) Some cell doublets appear empty (green arrow), but others (pink arrow) present darker, condensed material consistent in shape and location with a nucleus (white arrows). (D) Dark, condensed, and elongated material with morphological characteristics of metaphase chromosomes. The limit of the cell lacuna is visible (black arrow). (E) Caudal view of a juvenile emu skull (∼8–10 months old) showing the So and exoccipitals (Exo) in articulation. (F, G) Ground section (stained with Toluidine blue) of calcified cartilage from this emu skull showing cell doublets (pink arrows) with remnants of nuclei (white arrows) and others without intracellular content (green arrow).

A nesting ground yielding dozens of disarticulated nestlings assigned to the herbivorous duck-billed dinosaur *Hypacrosaurus stebingeri* was discovered in the 1980s in the Upper Cretaceous (Campanian) Two Medicine Formation of northern Montana (Museum of the Rockies, MOR 548, locality TM-066, Supplementary Text 1) [1,2]. Several limb and skull elements of these nestlings were subjected to microscopic analyses to answer growth-related questions [1] and to describe different types of cartilage [3]. The calcified cartilage seen within a supraoccipital in ground-section was especially interesting (Fig. 1B–D). Within the chondro-osseous junction (i.e., the part of the growth plate where bone replaces cartilage) closest to the left exoccipital, the tissues presented excellent microscopic preservation, such that cartilage could be distinguished from bone by exhibiting a translucent, amorphous extracellular matrix (ECM) and round, hypertrophic chondrocyte lacunae (Fig. 1B). At higher magnification, cellular structures still sharing a single lacuna (i.e., a cell doublet) [4,5] were seen, consistent with chondrocytes at the end of mitosis (Fig. 1C, pink arrow; Supplementary Fig. 1). Although many lacunae appear empty (Fig. 1B and C, green arrow), other lacunae (pink arrow) contain a material distinct from the matrix, including a darker material consistent in shape and location with a nucleus (Fig. 1C, white arrows). This is comparable to features of extant calcified cartilage [4] observed in ground sections of defleshed, juvenile emu skulls, where some lacunae are empty, and others retain cells and intracellular contents including nuclei (Fig. 1G).

Near the cell doublet (Fig. 1C), other microscopic structures consist of dark, condensed and elongated material, aligned along a plane and slightly mirroring each other (Fig. 1D). The cell lacuna surrounding these structures is visible (Fig. 1D, black arrow), but is even clearer under a different light setting (with a condenser, Supplementary Fig. 1). This dark material shares microstructural features with condensed chromatin, more precisely of chromosomes in metaphase of the cell cycle [6]. Similar chromosome-like structures have been observed in a fossil fern from the Jurassic [7], but the present study reports this type of exceptional microscopic preservation, at the sub-cellular level, in a fossil vertebrate and validates the observations with biochemistry.

We hypothesized that this exceptional morphological preservation would extend to the molecular level when methods commonly used to identify molecular and chemical markers in extant cartilage were applied to these fossil tissues. To test this hypothesis, we investigated molecular preservation of *Hypacrosaurus* cartilage at the extracellular, cellular and intracellular levels in another supraoccipital from the same nesting ground (Fig. 1A), similar in size to the one in which these structures were originally observed (Fig. 1B–D). This study specimen had not been previously embedded in resin. We capitalized on distinct chemical differences between cartilage and bone within this second *Hypacrosaurus* supraoccipital (Fig. 1A), and used the supraoccipitals of juvenile emus (*Dromaius novaehollandiae*) as extant homologues.

## RESULTS AND DISCUSSION

### Histology

During ontogeny, the supraoccipital arises as a primary cartilage which is then replaced by bone via endochondral ossification [3]. Growth occurs mostly at the chondro-osseous junction, where chondrocytes undergo cell division and hypertrophy. Chondrocytes encased in lacunae secrete ECM components including collagen II and glycosaminoglycans [4,8]. Conversely, osteoblasts and osteocytes secrete collagen type I, minimal amounts of glycosaminoglycans [9], and non-collagenous proteins [10].

Hypertrophic chondrocytes of the chondro-osseous junction have different fates (Supplementary Text 2) but many undergo cell death [4,5,11], eventually resulting in empty lacunae (e.g., Fig. 1G) followed by replacement with bone [4,12]. Even though one fossil cell preserves structures morphologically consistent with mitotic chromosomes in metaphase (Fig. 1D), we propose, based upon living comparisons, that it is not undergoing normal cell division, but rather presents the characteristics of a hypertrophic chondrocyte undergoing an early stage of cell death called chondroptosis [11,13,14] (Supplementary Fig. 1; Supplementary Text 2, 3). In this type of abnormal mitosis, specifically identified in senescent hypertrophic chondrocytes of avian growth plates, the DNA condenses and chromosomes adopt a metaphase-like arrangement [13–15] (Supplementary Text 2).

### Histochemistry

**Figure 2.**
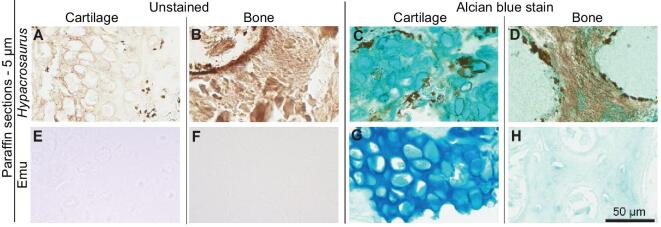
Alcian blue histochemical stain capitalized on differential presence of glycosaminoglycans in the calcified cartilage and bone of *Hypacrosaurus*. Unstained (A, B, E, F) and Alcian-blue stained (C, D, G, H) paraffin sections of *Hypacrosaurus* and emu cartilage and bone. A strong, positive blue staining is seen in *Hypacrosaurus* cartilage (C), comparable to the intense, but darker stain found in modern emu cartilage (G). This suggests that glycosaminoglycans are still present in the cartilaginous matrix of this dinosaur. In contrast, the fossil and extant bones show a very light blue stain (D, H). Images are at the same scale.

The ECM of extant cartilage and bone can be differentiated using Alcian blue, a histochemical stain that reacts intensely with acidic materials and much less so with basic ones. Cartilage incorporates acid mucins and glycosaminoglycans not found in bone [4,16]. Alcian blue was previously employed to differentiate tissues in *Tyrannosaurus rex* [16] and *Yanornis martini* [17]; we applied it here to paraffin sections of demineralized *Hypacrosaurus* cartilage and bone. Fossil (Fig. 2C) and extant (Fig. 2G) cartilage both demonstrated intense staining when compared to stained demineralized bone from the same organisms (Fig. 2D, H), supporting chemical differentiation between dinosaur tissues similar to that seen in extant tissues, and suggesting preservation of the original chemistry in these ancient tissues.

### Immunofluorescence and Immunohistochemistry

**Figure 3.**
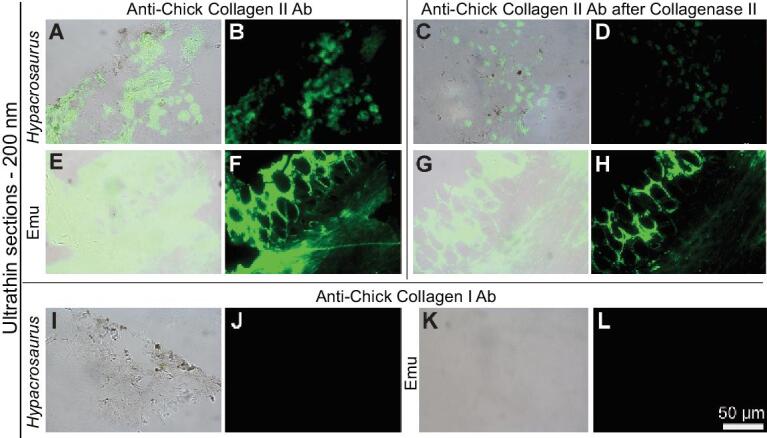
Immunohistochemical staining of *Hypacrosaurus* cartilage. (A, C, E, G, I, K) are overlay images showing cartilage and localized binding; (B, D, F, H, J, L) are fluorescent images using FITC label. *Hypacrosaurus* calcified cartilage (A, B) shows positive, localized staining when exposed to antibodies (Ab) raised against avian Collagen II, with green fluorescent signal representing antibody-antigen complexes arranged globularly in the extracellular matrix. Immunoreactivity is diminished, as illustrated by longer integration time and fainter signal, when compared to calcified and hyaline cartilage from an emu (E, F). Antibody reactivity was decreased after collagenase II digestion in *Hypacrosaurus* (C–D) and emu cartilage (G–H), demonstrating that reactivity to Collagen II is specific for epitopes of that protein. *Hypacrosaurus* (I, J) and emu cartilage (K, L) shows no staining when exposed to antibodies rained against avian Collagen I. Images are at the same scale.

Immunohistochemistry supports the persistence of protein epitopes consistent with collagen II (Fig. 3), the most abundant protein comprising the ECM of extant cartilage [4]. Ultra-thin sections of demineralized *Hypacrosaurus* cartilage were exposed to antibodies raised against avian collagen II. Green fluorescence on tissues reflects the location of antibody-antigen complexes (Fig. 3B). In extant emu cartilage, binding is present throughout the entire matrix (Fig. 3F). Binding in fossil cartilage is represented by a more globular pattern (Fig. 3B), suggesting that preserved collagen II epitopes are not homogenously distributed in the ECM of this *Hypacrosaurus*. Furthemore, immunoreactivity in *Hypacrosaurus* cartilage is diminished when compared to emu (Fig. 3F), as illustrated by longer integration times and fainter signal. This may indicate that fewer epitopes are preserved in the ancient tissues, or alternatively, that there is more phylogenetic distance between the dinosaur proteins and chicken proteins used to generate the antiserum. If the latter, not all epitopes would have been present in the living dinosaur.

To account for the possibility of non-specific binding, we digested the fossil and extant tissues with collagenase II, an enzyme specific for collagen II [18]. At the same integration times and parameters as in non-digested tissues, antibody reactivity was significantly decreased after digestion of both extant (Fig. 3H) and fossil tissues (Fig. 3D); providing further support for the presence of collagen II, and confirming the specificity of antibodies used in this study. As an additional specificity control, fossilized cartilage was exposed to antibodies raised against avian collagen I, the dominant protein in bone [10]. Because extant primary cartilage does not usually express collagen I [4], no binding was expected, and none was observed in either *Hypacrosaurus* (Fig. 3J) or emu cartilage (Fig. 3L). To control for non-specific binding of the secondary antibody or fluorescent label, primary antibodies were omitted, while keeping all other conditions identical; no cross-reactivity was observed (Supplementary Fig. 2). The most parsimonious explanation for these results is that epitopes of collagen II are preserved in this 75 million year-old dinosaur. Collagen II is not produced by microbes; positing a microbial source is not parsimonious or congruent with the data.

### Isolation of chondrocytes and DNA assays

**Figure 4.**
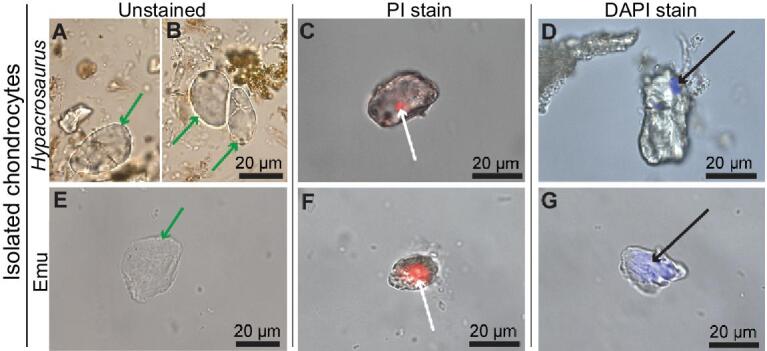
Isolated chondrocytes of *Hypacrosaurus* and their positive response to two DNA assays. (A, B, E) Isolated chondrocytes of *Hypacrosaurus* and emu photographed under transmitted light (green arrows). *Hypacrosaurus* chondrocytes were successfully isolated as individual cells (A) and cell doublets (B). *Hypacrosaurus* (C) and emu chondrocytes (F) showing positive response to propidium iodide (PI), a DNA intercalating dye, to a small and circular region that locates intracellularly (white arrows). *Hypacrosaurus* (D) and emu chondrocytes (G) also show a similar binding when exposed to 4^′^,6^′^-diamidino-2-phenylindole dihydrochloride (DAPI), another DNA-specific stain (black arrows) although in both cases, emu cell staining is significantly greater than in the dinosaur cells.

Although osteocytes have previously been isolated from dinosaur bone [19,20], here, we show the first isolated dinosaur chondrocytes (Fig. 4). Unlike dinosaur osteocytes that often present a reddish hue due to iron inclusions [21], *Hypacrosaurus* chondrocytes are transparent (Fig. 4A and B), suggesting a different preservation mode. They lack filopodia and present the round morphology consistent with all vertebrate chondrocytes [22] (including emu, Fig. 4E), but distinct from the fusiform shape of osteocytes [20]. A few cell doublets were also isolated in *Hypacrosaurus* (e.g., Fig. 4B).

Although the cells appeared empty under transmitted light (as do emu chondrocytes) (Fig. 4A and B), we tested these microstructures for the presence of chemical markers consistent with DNA [20] using two complementary histochemical stains, propidium iodide (PI) [23] and 4^′^,6^′^-diamidino-2-phenylindole dihydrochloride (DAPI) [24]. PI intercalates between every four to five base pairs of double-stranded DNA, with little or no sequence preference, and is detected in red frequencies when stimulated by fluorescent light [23]. PI does not stain DNA in a living cell, but only in dead cells. Therefore, positive PI staining cannot arise from contamination with living (i.e., microbial) cells [25]. DAPI binds preferentially to double-stranded DNA in both living and dead cells. It is sequence dependent requiring at least three successive AT base pairs as a binding site [24].

Specific staining of both PI (Fig. 4C) and DAPI (Fig. 4D) is observed inside the isolated cartilage cells of *Hypacrosaurus*, following the pattern seen in extant cells (Fig. 4F and G), but diminished in the ancient ones. This not only supports that the compound within these cells is chemically consistent with DNA, but that material is double stranded, and of a minimum length of 6 base pairs [24]. These molecules must be reduced in concentration relative to the extant ones because of the greatly reduced area stained; this pattern was also observed with dinosaur osteocytes (in *Tyrannosaurus rex* and *Brachylophosaurus canadensis*) [20]. Typical taphonomic alteration of DNA involves, among other processes, backbone breakage [26], a phenomenon expected to have occurred in cells of this antiquity. Additionally, because the PI stain in the fossil cells localizes to such a small area, it may indicate that the nuclear material was in a condensed state at the time of death. DNA condensation occurs naturally during mitotis, interphase, and all types of cell death (Supplementary Text 2) [27,28]. Because the isolated cells of *Hypacrosaurus* come from the chondro-osseous junction, where most chondrocytes eventually die, it is possible that the cells showing PI and DAPI staining (Fig. 4C and D) were senescent (e.g., chondroptotic, or apoptotic cells), with condensed nuclear material. Extant senescent chondrocytes can show different morphologies of condensed nuclear material (with or without a fragmented nuclear membrane), such as condensed chromatin granules, a chromosome-like arrangement, or multiple apoptotic condensations (Supplementary Text 2, 3).

An alternative hypothesis, that the staining arises from microbial contaminant, is not supported (*contra* [25,29]); there is no mechanism for exogenous DNA to penetrate an intact membrane and localize to a single point specifically inside the cell, demonstrating no reactivity in any other region. We caution that only the sequencing of the material reactive to both PI and DAPI can confirm that it is dinosaurian in origin, however the combined data at the histological, cellular and molecular levels (Figs 1–4) robustly support the hypothesis that the cartilage of *Hypacrosaurus* has remnants of original chondrocytes, original nuclear material, and endogenous compounds chemically consistent with DNA. In addition, these data support that structures morphologically consistent with nuclei and chromosomes, first seen in petrographic ground sections (Fig. 1; Supplementary Fig. 1) are endogenous to this dinosaur. Although it has been suggested that similar, cell-like structures recovered in dinosaur bones could be the result of biofilm infiltration [25], the pattern of reactivity observed when biofilm was exposed to DAPI and PI staining during a previous study [20] is inconsistent with the one observed here. It is reasonable and logical to propose that fossil dinosaur bone contains contaminating microbial communities [29], but the specific case that we present here, where isolated chondrocytes show intracellular reactivity with PI and DAPI (Fig. 4), does not match the staining pattern of ‘cell clusters’ of contaminating biofilms [29].

We have shown that cartilage found in MOR 548 is extremely well preserved, not only at the histological level with an unreported, sub-cellular type of preservation (Fig. 1; Supplementary Fig. 1), but also at the cellular and the molecular levels (Figs 2–4). This study provides the first clear chemical and molecular demonstration of calcified cartilage preservation in Mesozoic skeletal material, and suggests that in addition to cartilage-specific collagen II, DNA, or at least the chemical markers of DNA (for example, chemically altered base pairs that can still react to PI and DAPI), may preserve for millions of years.

The assumption of a temporal limit on molecular longevity has hindered the pursuit of molecular data from fossils older than ∼1 million years (MA). A short temporal range is predicted for informative biomolecules (∼1 MA for proteins, and ∼100,000 years for DNA; with 700,000 years as the oldest genome report) [30–32]. However, these assumptions have been challenged by multiple studies on Mesozoic fossil remains reporting evidence of chemical and organic remnants, including extracellular proteins and pigments (e.g., [33–37]), cytoskeletal proteins [20], compounds that localize to cell interiors that are chemically consistent with DNA [20; and the present study] and peptide sequence data including histone proteins, a protein not found in bacteria [38–40].

Here, data derived from dinosaurian calcified cartilage add to the growing evidence across specimens and biomolecules that original, endogenous organics persist into deep time under exceptional conditions. Interestingly, all the materials collected at this nesting ground were disarticulated, suggesting that a phenomenon other than rapid burial allowed such exquisite preservation (Supplementary Text 1). Other juvenile hadrosaur material from the Two Medicine formation showed exquisite preservation of cartilage [41]. Calcified cartilage may represent a better candidate than bone for biomolecule preservation, because it exhibits multiple factors that contribute to molecular stabilization [42]. These include an ECM without vascularization, making it less porous, with less surface area available for ground water and microbes that result in biodeterioration; an ECM with a higher mineral:organic ratio than that of bone [43]; and hypoxia [44]. Oxygen levels are lowest specifically in the calcified, hypertrophic chondrocyte zone, which may serve to sequester cells, preventing oxidative damage.

## CONCLUSION

The identification of chemical markers of DNA in *Hypacrosaurus* suggest it may preserve much longer than originally proposed [30,31]. Even though it is clear that contamination does exist in fossil material and complicates identifications of original organic molecules, it can be accounted for with proper controls. Contamination is not a plausible explanation in this case, and to this date, the possible preservation of original proteins and DNA in deep time has not been convincingly eliminated with data. Although extensive research and sequencing is required to further understand DNA preservation in Mesozoic material, along with its chemical and molecular alterations, our data suggest the preserved nuclear material in *Hypacrosaurus* was in a condensed state at the time of the death of the organism, which may have contributed to its stability. We propose that DNA condensation may be a favorable process to its fossilization. Additionally, as was suggested for protein fossilization [20,45,46], crosslinking may be another mechanism involved in the preservation of DNA in deep time.

## METHODS

All assays using fossil material were conducted in lab facilities dedicated to fossil analyses, using aseptic techniques, and did not at any time come in contact with homologous tissues from emu used as positive controls. The latter were analyzed in a separate lab, using dedicated instruments and solutions.

### Fossil material

We used two disarticulated supraoccipitals (bones of the basicranium) of *Hypacrosaurus stebingeri* (MOR 548) that were similar in size and preservation state. The first was embedded in polyester resin and prepared at the MOR in the 1980s (Fig. 1B–D). Because resin interferes with some molecular methods, a second non-embedded supraoccipital (Fig. 1A) from the same fossil site was chosen to perform chemical and molecular analyses (Figs 2–4). Based on their size, these elements were estimated to belong to nestlings with a skull length of about 20 cm, and an overall full length of 2 m [1,3]. Adults of this species usually reach a full length of 9 m [47].

### Extant material

We used a total of four juvenile emus (*Dromaius novaehollandiae*) donated cadaveric to the MOR by Montana Emu Ranch, between a few weeks and a few months post-hatching (between 8–10 months). Two were kept frozen, and two were sent to a dermestid beetle colony and defleshed for other studies on emu skulls [48] (in emu skulls, most of the hyaline, unmineralized cartilage is eaten by beetles, leaving mostly calcified cartilage). Data for the ground section (Fig. 1E–G) is from the emu skull MOR OST 1802 [48]. Histochemical, immunological and DNA staining were performed on the other three emu specimens at the supraoccipital-exoccipital junction (homologous to the calcified cartilage region of interest in *Hypacrosaurus*).

### Ground sections

One supraoccipital of MOR 548 was embedded whole in polyester resin (according to standard methods) [49]. A relatively thick slice taken using a Buehler Isomet 1000 precision saw was attached to a glass slide with epoxy and ground to desired thickness (100 microns) with a Buehler Ecomet Grinder. The slide (Fig. 1B–D) was studied by light microscopy under transmitted light with a Nikon Optiphot-Pol polarizing microscope. Photographs were taken with a Nikon DS-Fi1 digital sight camera and the NIS ELEMENTS BR 4.13 software.

For comparison with extant emu material (Fig. 1E–G), fragments including basicranial cartilaginous joints (synchondroses) from the skull of MOR OST 1802 were extracted with a dremel and diamond blade, fixed in 10% neutral buffered formalin (NBF), and further prepared for embedding and stained with Toluidine blue (Supplementary Methods).

### Histochemistry/Alcian blue staining

Fragments taken from the periphery of the supraoccipital of MOR 548 were chosen to optimize chances of obtaining mostly cartilage, rather than bone. Emu samples also included calcified cartilage from the supraoccipital-exoccipital synchondrosis, as well as some underlying bone.

These fragments from extant emu were fixed with 10% NBF overnight, then demineralized in 500 mM EDTA (disodium ethylenediaminetetraacetic acid) (pH 8.0) until all mineral was removed, and again subjected to fixation as above. Because of the fragile nature of demineralized fossil tissues, MOR 548 samples were embedded in 3% agar (Becton Dickinson Cat# 214530) to stabilize the tissues prior to sectioning. Extant tissues and agar embedded tissues from MOR 548 were then subjected to routine preparation for paraffin histology, embedded in paraffin, cut on a microtome at 5 microns, and stained with Alcian blue (Supplementary Methods).

### Immunofluorescence/Immunohistochemistry

Fossil fragments were first embedded in 3% agar (Becton Dickinson Cat# 214530), then demineralized in EDTA (0.5 M, pH 8.0) for two weeks, washed with 1× phosphate buffered saline (PBS) and prepared for further embedding and sectioning for analyses (Supplementary Methods). In a separate laboratory, cartilage tissues were sampled from a dry emu skull fragment (MOR-OST 1800), fixed for one hour at room temperature in 10% NBF (Supplementary Methods).

Sections (200 nm) were cut on a Leica EM UC6 Ultramicrotome and exposed to a Rabbit anti-chicken collagen type II (Abcam ab21290) primary antibody (Supplementary Methods). For antibodies raised against avian collagen I, we used the antibody US Biological C7510–13B. Sections were also incubated in antibody dilution buffer only, to which no primary antibodies were added, to control for spurious binding of the secondary antibody (Supplementary Fig. 2).

### Isolation of chondrocytes and DAPI/PI Staining

Fragments were demineralized in EDTA (0.5 M, pH 8.0) for at least 2 weeks with daily changes. Resulting debris after demineralization were collected from the bottom of the wells and centrifuged for 2 min at 400 rcf. After removing the supernatant, the pellets were re-suspended in 1× PBS to remove the remaining EDTA and the process was repeated three times. After final centrifugation, the pellets were incubated with the iron chelator pyridoxal isonicotinoyl hydrazone (PIH) solution (10 mM PIH in 50 mM NaOH [50]) overnight at room temperature, then washed with 1× PBS and centrifuged to form cell pellets, used for DAPI/PI staining (Supplementary Methods).

Emu cartilage layers were excised from the supraoccipital-exoccipital synchondrosis of a young emu (but more cartilage was needed so femoral cartilage was excised as well), incubated in 0.1% Sodium Azide for 1.5 hours then cut into 0.5–1 mm slices with a sterile razor blade. Slices were then digested with 3 mg/mL collagenase type II (Worthington CLS-2) in Dulbecco's phosphate-buffered saline (D-PBS) pH 7.2 with 0.1% Sodium Azide at 37°C overnight or until the majority of the cartilage matrix was removed. The samples were centrifuged at 300 rcf for 5 min to pellet cells. Cells were re-suspended in 10% NBF (pH 7.2–7.4) at room temperature for 30 min to fix, then centrifuged as before, and washed twice with PBS. Cells were pelleted and subjected to DAPI/PI staining (Supplementary Methods).

## Supplementary Material

nwz206_Supplemental_File
