# Supplementary material for: Evidence of proteins, chromosomes and chemical markers of DNA in exceptionally preserved dinosaur cartilage
Source: Natl Sci Rev. 2020 Jan 12;7(4):815–22. doi: 10.1093/nsr/nwz206 (PMC8289162; doi:10.1093/nsr/nwz206)
Supplement: nwz206_Supplemental_File [file nwz206_supplemental_file.docx]

**SUPPLEMENTARY INFORMATION FOR**

**Evidence of proteins, chromosomes and chemical markers of DNA in exceptionally preserved dinosaur cartilage**

Alida M. Bailleul, Wenxia Zheng, John R. Horner, Brian K. Hall, Casey M. Holliday, Mary H. Schweitzer

correspondence to: alida.bailleul@ivpp.ac.cn

**This PDF file includes:**

Supplementary Methods

Supplementary Text

Supplementary Figures 1-2

Supplementary Methods

**Toluidine blue staining of the emu ground-section**

The fragment of MOR OST 1802 was fixed in 10% NBF, transferred to 70% ethanol, dehydrated through in graded ethanol series, cleared in xylene, and embedded in Buehler Epothin 2 resin. A relatively thick slice taken from Buehler Isomet 1000 precision saw was attached to a plexiglass slide with cyanoacrylate glue and ground to desired thickness (100 microns) with a Buehler Ecomet Grinder. The slide was etched in 1% formic acid for 30 seconds, rinsed with water, stained with a solution of Toluidine blue (1% in Phosphate buffer solution pH 8.0) at 57°C for 5 minutes then rinsed again in water (this method is already described in [1]. It was imaged with the same system as that listed for MOR 548 in the main manuscript.

**Histochemistry and Alcian blue staining**

Extant tissues and agar embedded tissues from MOR 548 were then subjected to routine dehydration (via sequential incubations in 70%, 80%, 90%, 95%, and 100% ethanol for ~ 1 hour each). The tissue blocks were then placed in an additional two 100% ethanol solution to ensure that all the water was removed, followed by three 30 min incubations in xylene to clear the tissues. Tissues were then transferred to melted paraffin, three separate sequential incubations for 30 min each to complete infiltration, then embedded in paraffin wax (Paraplast Plus EMS Cat#19216) for sectioning. Sections were cut at 5 microns on a rotary microtome (either a Leica RM 2255 or a ThermoFisher Shandon Finesse Me+), placed in a warm water bath at 44°C with section adhesive (Sta-on Surgipath, Leica), mounted on charged slides (Superfrost Plus, Fisher Scientific), then dried in an oven at 60°C for one hour.

Alcian blue, a stain used to differentiate and diagnose cartilage in extant materials was applied to dinosaur and emu bone and cartilage as follows: Paraffin sections were deparaffinized with xylene, dehydrated through a graded ethanol series (70%, 80%, 90%, 95%, and 100% ethanol), oxidized in 1% H_5_IO_6_ (periodic acid) for 10 min, rinsed under running tap water, then exposed to Alcian blue 8GX (1% in 3% acetic acid) for 30 min, rinsed again, then dehydrated with a graded ethanol series, followed by several incubations with 100% xylene. Mounting medium (Permount, Fisher Scientific) and cover glass were applied for visualization.

Negative control (No staining): Sections of bone and cartilage of MOR 548 and emu were treated as above; i.e., demineralized fragments were embedded in either paraffin, or agar and paraffin, sectioned and dried. Paraffin sections were deparaffinized with xylene. Mounting medium (Permount, Fisher Scientific) and cover glass were applied for visualization.

**Immunofluorescence/Immunohistochemistry**

Demineralized MOR 548 or fixed extant specimens were dehydrated in 70% ethanol for two 30 min incubations, and then infiltrated with a 2:1 mixture of LR White (EMS Cat #14383, hard grade): 70% ethanol for one hour. Samples were then incubated in 2 changes of 100% LR White, a polymer designed to be water permeable, therefore optimal for immunohistochemical studies, for 1 hour each time. Specimens were placed in gelatin capsules, covered With LR White embedding medium, sealed, and polymerized at 60ºC for 24 hours.

Sections (200nm) were cut on a Leica EM UC6 Ultramicrotome, transferred to six-well, Teflon-coated slides, and dried thoroughly. Sections were etched with Proteinase K (PCR grade, Roche, 25 µg/ml) in 1x PBS buffer at 37ºC, followed by two incubations in 500 mM EDTA (pH 8.0) for epitope retrieval, and two incubations in 1 mg/ml sodium borohydride for 10 min each for to reducing autofluorescence. All incubations were separated by sequential washes (two times for five minutes each) in PBS. Sections were then incubated for two hours with 4 % normal goat serum in PBS to occupy non-specific binding sites and prevent spurious binding. Rabbit anti-chicken collagen type II (Abcam ab21290) primary antibody diluted to 1:75 in primary dilution buffer (1% Bovine Serum Albumin (BSA) (Fisher, BP1660-100), 0.1% Cold Fish Skin Gelatine (Sigma G7765), 0.05% Sodium Azide (Sigma S-8032), 0.01M PBS pH 7.2) then applied to sectioned tissues and incubated overnight at 4ºC. Sections were also incubated in antibody dilution buffer only, to which no primary antibodies were added, to control for spurious binding of the secondary antibody (Fig. S2). All sections were washed thoroughly to remove unbound antibody, then incubated with secondary antibody (Biotinylated Goat Anti-Rabbit IgG (H+L) (Vector BA-1000; diluted 1:500)) for two hours at room temperature. Fluorescein Avidin D (FITC, Vector Laboratories A-2001) diluted 1:1000 was applied to all sections and allowed to bind for one hour at room temperature. All incubations were separated by sequential washes (two times for 10 min each) in PBS w/Tween 20 (ACROS Organics) followed by two 10 min-rinses in PBS. Finally, the sections were mounted with Vectashield Anti-Fade mounting medium (Vector H-1000), and coverslips were applied. The sections were examined with a Zeiss Axioskop 2 Plus biological microscope and captured using an AxioCam MRc 5 (Zeiss) with ×10 ocular magnification; data were collected using the Axiovision software package (version 4.7.0.0).

**PI and DAPI staining**

Isolated MOR 548 and emu chondrocytes (in separate labs) were incubated with 200μl of 0.5% Triton X100 in PBS for 10 min, then centrifuged at 300 rcf for 2 min to form cell pellets. Supernatant was discarded and cell pellets were re-suspended, washed with PBS, and re-pelleted. Part of the cells was incubated with 40μl propidium iodide (PI) solution (BD Biosciences 556463) for 30 min at room temperature in the dark. Additional cells were stained with 300mM DAPI dihydrochloride (Invitrogen D21490) in Citric acid-Sodium phosphate buffer pH 7.0 (0.1M citric Acid, 0.2M Disodium hydrogen phosphate Na_2_HPO_4_) for 10 min. To remove excess stain, each sample was centrifuged, and washed 3 times with PBS. Cells were then transferred to 6-well "PTFE" Printed Slides, and Vectashield H-1000 Mounting medium and coverslips were applied. Images were obtained in the dark using a Zeiss AxioSkop2 Plus fluorescence microscope.

**Supplementary Text 1:**

**Brief notes on the geological setting and mode of tissue preservation at the MOR nesting site (TM-066)**

In this Upper Cretaceous (Campanian) hadrosaur nesting ground from the Two Medicine Formation, dozens of disarticulated nestling skeletal elements of *Hypacrosaurus stebingeri* were found [2, 3]. Bones were preserved in a fine-grained siltstone, interpreted as a small rivulet that had transected a nesting ground [2]. The siltstone layer was sandwiched between layers of mud rock. Burial, apparently, was not particularly rapid, because most of the nestling bones were disarticulated. It is interesting to see such exquisite preservation at the histological, cellular, subcellular, and molecular level (see main manuscript) in this material, as apparently a process other than rapid burial allowed such preservation. Microbes may have also played a role in the rapid precipitation of carbonates on these organic remains, as it has been demonstrated frequently in other settings (e.g., [4] and references therein).

Cartilage, in general, is not predicted to survive well over geological time at the molecular level, because: 1) most of the extracellular matrix (ECM) of cartilage is unmineralized (i.e., hyaline) in life ; 2) the ECM contains high amounts of water, and 3) the ECM is often located at extremities (e.g., growth plate cartilage or articular cartilage) in direct contact with sediments, ground water and environmental microbes, all of which contribute to molecular degradation. However, when cartilage is calcified, it may be more susceptible to cellular and molecular preservation even than bone (see arguments in main manuscript). Exceptional preservation in the calcified cartilage of juvenile *Maiasaura*, another hadrosaur from the Two Medicine Formation, was also observed using elemental analysis and scanning electron microscopy [5]. The authors showed that the ECM of the calcified cartilage in juvenile *Maiasaura* had the same Ca:P atomic percent ratios as that of calcified cartilage of extant birds, and concluded that the original biomineral (hydroxyapatite) had been preserved for millions of years [5]. More taphonomic, histological, and molecular data on additional material and cartilage from the Two Medicine Formation will be require necessary to understand fully the settings that favored this exceptional type preservation through deep-time.

**Supplementary Text 2:**

**Notes on the fate of hypertrophic chondrocytes and types of cell death at the chondro-osseous junction of avian growth plates.**

All cells eventually die by two main cellular death processes: 1) necrosis, which is the natural process of physiological cell death/senescence [6]; and 2) apoptosis, which is programmed cell death [7, 8]. Individual chondrocytes die by necrosis and apoptosis, but also through other types of cell death [9, 10] (discussed below).

As stated in the main manuscript, chondrocytes become hypertrophic as they approach the chondro-osseous junction of growth plates found at the ends of long bones, or at basicranial synchondroses (the latter case consisting of two growth plates back to back [1, 5]). For decades, it was thought that all hypertrophic chondrocytes undergo apoptosis, resulting in empty lacunae that are invaded by bone cells at the ossification and vascular front. However, mounting evidence has shown that this is erroneous in two main aspects: 1) many hypertrophic chondrocytes do not die, but instead dedifferentiate into osteoblast progenitor cells, reinitiate cell division, and change phenotype, differentiating into osteoblasts [11-15]; and 2) many of the presumed apoptotic chondrocytes in fact do not present **all** of the typical, classical cellular features originally identified in apoptosis [7, 8]. Classical apoptotic cells have an unmistakable morphology, mostly visible using transmission electron microscopy (TEM; but also detectable histologically, e.g., see Figure 2a in [9]): the nuclear chromatin condenses into dark crescents, caps or spheres, the cell shrinks and buds into circular apoptotic bodies (which are membrane-bound), and the bodies are rapidly phagocytosed extracellularly [9].

It has been noted that in past studies many hypertrophic chondrocytes were erroneously identified as apoptotic (see examples listed in [9]), because they presented **a mix** of the features of both apoptosis and necrosis. In necrosis, apoptotic bodies do not form and the cell usually swells (instead of shrinking; [6]). This newly discovered type of chondrocyte death was named ‘chondroptosis’ [9].

Chondroptosis was originally identified in avian hypertrophic chondrocytes as a type of aberrant cell death [16], but was not named chondroptotis until later in a study that also reported this process in mammalian chondrocytes [9]. The process was identified in a cell population called ‘dark chondrocytes’, because they appear darker than other chondrocytes in TEM [16]. In these dying chondrocytes, it was noted that the DNA condensed (as in all other types of cell death, including apoptosis; [10]), but that classical apoptotic bodies did not form. The nuclear chromatin was enclosed in an intact, or partially lysed nuclear membrane, and condensed in the periphery into small granules. These cells therefore show a dense granular karyoplasm [16] (the karyoplasm is the substance forming the nucleus, enclosed by the nuclear membrane).

In some instances, the density of this karyoplasm increased and in other non-peripheral areas of the nucleus, the chromatin condensed into chromosome-like structures that adopted a prometaphase, or metaphase arrangement (Figs. 4d-e in [16], also in [17]). Even though the chromatin is adopting a metaphase arrangement (which would correspond normally to a mitotic cell stage), it does not represent normal mitosis, but instead a type of ‘abortive mitosis’, or ‘mitotic catastrophe’ taking place in this aberrant type of cell death [16, 17]. This metaphase arrangement had also been identified previously in ‘ageing’ avian chondrocytes by the same first author [18], which cells were later determined to be dark chondrocytes [16]. Additional characteristics of cartilage cells undergoing chondroptotis are cell shrinkage within the lacuna, followed by a secretion of intralacunar matrix [10]. At the end of chondroptosis, the chondrocyte is degraded and disappears through autophagy, eventually resulting into an empty lacuna [9].

**Supplementary Text 3:**

**Additional description/identification of the chondroptotic cell in *Hypacrosaurus***

Based on the cellular characteristics and types of cell death described above in avian chondrocytes (Supplementary Text 2), we can identify the cell presenting chromosome-like structures in *Hypacrosaurus* (Fig. 1D) as chondroptotic [9, 16, 17]. Even though the chromosomes are arranged as if they are in metaphase, the cell is not dividing because it is found within an already calcified matrix. Note that the two cells part of the cell doublet have already finished cell division, because they show two individualized plasma membranes (Fig. S1, left pink arrow).

Because petrographic ground sections of fossil tissues are dark and much thicker than cells (the slide thickness is about 100µm, but hypertrophic cartilage cells usually range between 15-20 µm in size), it is important to look at different planes of the sections with a slight change in focus, and with different light settings. Under transmitted light, the chromosomes are very clear (Fig. 1D), but the limits of the chondrocyte lacuna and plasma membrane are not. When the same area of calcified cartilage is observed under transmitted light paired with a condenser (Fig. S1), the limits of the lacuna are much more obvious.

Moreover, this micrograph (Fig. S1) shown at a slightly different plane (more towards the surface of the slide, in a more peripheral area, but still within the cell; whereas the chromosomes are deeper within the section and cell) shows additional characteristics of a chrondroptotic cell (Fig. S1):

1. The cell is slightly smaller than the rest of the cells, suggesting cell shrinkage occurred [9].
2. It is surrounded by a darker ECM, with apparent successive stages of matrix deposition as shown by faint concentric lines. These are not found around other cells, and is clearly distinct from both the interterritorial matrix (ITM) and the territorial matrix (TM) of the rest of the cartilage. It most likely represents the intralacunar matrix (ILM) deposited by shrunken chondroptotic cells [10].
3. It has an irregular plasma membrane (IPM; clearly visible in the lower left inset, a photograph showed with a slightly different focus; Fig. S1). IPMs are characteristics of chondroptotic cells, but not of apoptotic cells [9].
4. It presents numerous dark granules, some having a rod-like morphology. These granules present both the morphological features and the same size range of the chromatin granules reported in the granular karyoplasm of avian, dark chondrocytes [16, 18]. It is known that flocculation of the chromatin occurs in dying chondrocytes, in the periphery of the nucleus (not within the center of the nucleus), into rod-like granules, with a surface area twice that of younger chondroblasts (in younger chondroblasts, the granules were more circular and had a diameter of about 0.33µm; [18]).
5. The cell does not present a clear nuclear membrane, but in chondroptosis, the condensed nuclear material (i.e., the granular chromatin in the periphery of the nucleus, which may or may not be accompanied by a metaphase-like arrangement of the chromosomes more in the center of the nucleus) is sometimes enclosed into a partially dissolved nuclear membrane [16]. The nuclear membrane in MOR 548 may have been partially dissolved, or was present but simply not preserved/not visible here.

All of these characteristics support that this cell in *Hypacrosaurus* is a dying chondrocyte (Fig. 1D in the main manuscript, red arrow in Fig. S1), most likely homologous to the population of dark chondrocytes seen in avian growth-plates [16-18], which undergo chondroptosis. This cell does not present the characteristics of apoptosis: the intracellular structures clearly show a dark, sinuous, elongated material, consistent with chromatin (Fig. 1), incongruent in shape with apoptotic bodies, which are circular (e.g., see Fig. 2a in [9]). A metaphase arrangement and condensation of the chromatin is not seen in apoptosis, nor any other type of cell death (e.g., necrosis, or cells that died by ‘paralysis’; [10]). The dark intracellular structures (i.e., rod-like granules, and elongated material; Fig. 1, and Fig. S1) are also inconsistent in size and shape with calcifying matrix vesicles (i.e., cell derived, membrane enclosed units associated with hydroxyapatite deposition; [19]). Matrix vesicles are an order of magnitude smaller (see the TEM images in [20, 21]), they have been reported to range between 200 to 500nm [22].

Together, these arguments support our identification, that the elongated structures seen in one chondrocyte of MOR 548 (Fig. 1D) are indeed chromosomes, showing a metaphase arrangement, typical of hypertrophic chondrocytes undergoing an early stage of chondroptosis.


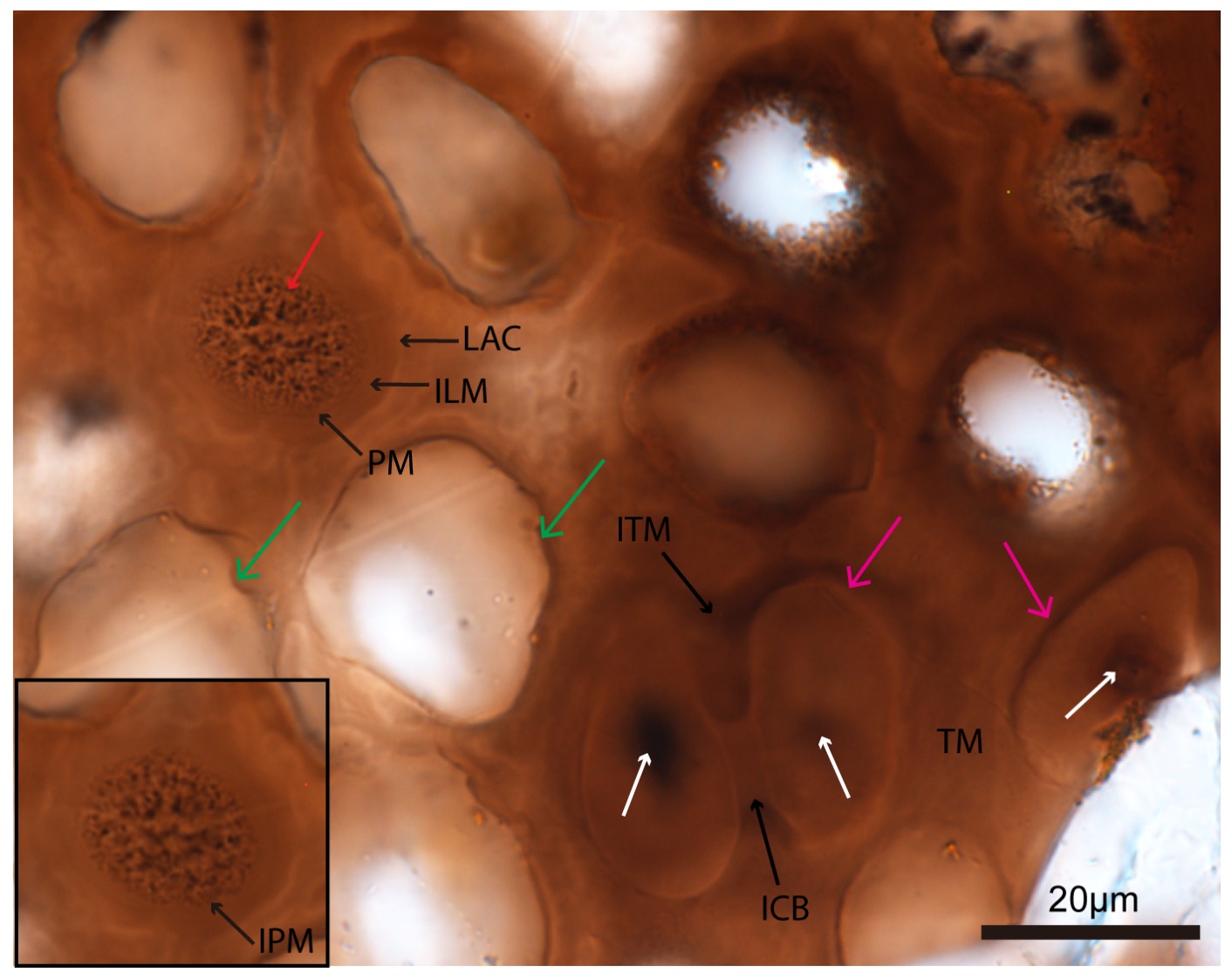


**Figure. S1. Photomicrograph of *Hypacrosaurus* (MOR 548) cartilage observed under transmitted light combined with a condenser.** This represents the same area shown in Figure 1 (main manuscript), at a slightly different plane of the section (more peripherally within the cell). Some lacunae are empty (green arrows), and others (pink arrows) have a condensed, circular and dark material consistent in location with a nucleus (white arrows). The cell doublet still has an intercellular bridge (ICB), but the two plasma membranes are individualized, meaning the cells have finished cell division and are in a late phase of cytokinesis. The one cell showing metaphase chromosomes (Fig. 1), at this more peripheral plane (but still within the cell), shows dark granules that are circular, to rod-like, consistent in shape and size with the dense granular karyoplasm (red arrow) reported in avian chondroptotic cells. The original limits of the lacuna (LAC) are clearly visible, and the cell has shrunk and secreted intralacunar matrix (ILM). The plasma membrane (PM) is roughly circular, but a slight change of focus on that cell (inset) shows it is irregular (irregular plasma membrane: IPM). IPMs are typical of chondroptotic cells.

Additional abbreviations: ITM: Intraterritorial matrix; TM: territorial matrix. The photomicrograph and the left inset are at the same scale.


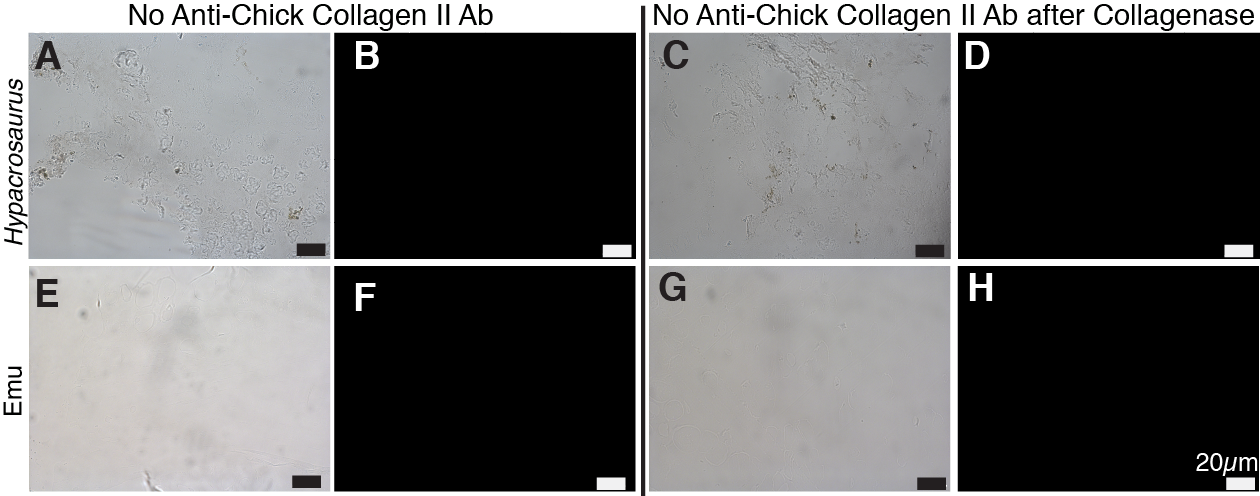


**Figure S2. Controls for immunohistochemical analyses, in which no primary antibody was applied, but all other parameters, including data collection parameters, were kept identical to test conditions, to control for false positives due to spurious binding of secondary antibody**. These images were taken from the same samples as presented in the main manuscript and in Fig. 3, at the same parameters; no reactivity to antibodies is demonstrated. A, C, E, G are overlay images; B, D, F, H are fluorescent images. A, B, C, D) *Hypacrosaurus* calcified cartilage (MOR 548); E, F, G, H) Emu calcified cartilage. Scale bar is 20μm and the same for all images.

**REFERENCES**

1. Bailleul AM, Horner JR: **Comparative histology of some craniofacial sutures and skull-base synchondroses in non-avian dinosaurs and their extant phylogenetic bracket**. *Journal of Anatomy* 2016, **229**(2):252-285.

2. Horner JR, Currie PJ: **Embryonic and neonatal morphology and ontogeny of a new species of *Hypacrosaurus* (Ornithischia, Lambeosauridae) from Montana and Alberta**. In: *Dinosaur eggs and babies.* Edited by K C, KE H, JR H. New York: Cambridge University Press; 1994: 312-336.

3. Varricchio DJ, Horner JR: **Hadrosaurid and lambeosaurid bone beds from the Upper Cretaceous Two Medicine Formation of Montana: taphonomic and biologic implications**. *Canadian Journal of Earth Sciences* 1993, **30**(5):997-1006.

4. Briggs DE: **The role of decay and mineralization in the preservation of soft-bodied fossils**. *Annual Review of Earth and Planetary Sciences* 2003, **31**(1):275-301.

5. Barreto C, Albrecht RM, Bjorling DE, Horner JR, Wilsman NJ: **Evidence of the Growth-Plate and the Growth of Long Bones in Juvenile Dinosaurs**. *Science* 1993, **262**(5142):2020-2023.

6. Sotres-Vega A, Villalba-Caloca J, Gaxiola-Gaxiola MO, Mendoza-Santiago M, Morales-Tiburcio JA, Hernández-Jiménez C, Figueroa-Cavero F: **Microscopic Evaluation of Necrotic Cell Death in the Cartilage Destined for Experimental Tracheal Allografts: Lyophilization vs Cryopreservation**. *Cryopreservation in Eukaryotes* 2016:29.

7. Elmore S: **Apoptosis: a review of programmed cell death**. *Toxicologic Pathology* 2007, **35**(4):495-516.

8. Kerr JF, Wyllie AH, Currie AR: **Apoptosis: a basic biological phenomenon with wideranging implications in tissue kinetics**. *British Journal of Cancer* 1972, **26**(4):239.

9. Roach H, Aigner T, Kouri J: **Chondroptosis: a variant of apoptotic cell death in chondrocytes?** *Apoptosis* 2004, **9**(3):265-277.

10. Roach H, Clarke N: **Physiological cell death of chondrocytes in vivo is not confined to apoptosis: new observations on the mammalian growth plate**. *The Journal of Bone and Joint Surgery British Volume* 2000, **82**(4):601-613.

11. Roach HI: **New aspects of endochondral ossification in the chick: chondrocyte apoptosis, bone formation by former chondrocytes, and acid phosphatase activity in the endochondral bone matrix**. *Journal of Bone and Mineral Research* 1997, **12**(5):795-805.

12. Roach HI, Erenpreisa J, Aigner T: **Osteogenic differentiation of hypertrophic chondrocytes involves asymmetric cell divisions and apoptosis**. *The Journal of Cell Biology* 1995, **131**(2):483-494.

13. Enishi T, Yukata K, Takahashi M, Sato R, Sairyo K, Yasui N: **Hypertrophic chondrocytes in the rabbit growth plate can proliferate and differentiate into osteogenic cells when capillary invasion is interposed by a membrane filter**. *Plos One* 2014, **9**(8):e104638.

14. Park J, Gebhardt M, Golovchenko S, Perez-Branguli F, Hattori T, Hartmann C, Zhou X, Stock M, Schneider H, von der Mark K: **Dual pathways to endochondral osteoblasts: a novel chondrocyte-derived osteoprogenitor cell identified in hypertrophic cartilage**. *Biology Open* 2015, **4**(5):608-621.

15. Tsang KY, Chan D, Cheah KS: **Fate of growth plate hypertrophic chondrocytes: death or lineage extension?** *Development, Growth & Differentiation* 2015, **57**(2):179-192.

16. Erenpreisa J, Roach HI: **Aberrant death in dark chondrocytes of the avian growth plate**. *Cell Death and Differentiation* 1998, **5**(1):60.

17. Erenpreisa J, Roach HI: **Aberrations of cell cycle and cell death in normal development of the chick embryo growth plate**. *Mechanisms of Ageing and Development* 1999, **108**(3):227-238.

18. Ērenpreisa J, Zhukotsky A: **Interphase genome as the active space: chromatin dynamics during chick embryo chondrogenesis**. *Mechanisms of Ageing and Development* 1993, **67**(1-2):21-32.

19. Lotz M, Hashimoto S, Kühn K: **Mechanisms of chondrocyte apoptosis**. *Osteoarthritis and Cartilage* 1999, **7**(4):389-391.

20. Bonucci E: **Fine structure of early cartilage calcification**. *Journal of Ultrastructure Research* 1967, **20**(1-2):33-50.

21. Anderson HC: **Electron microscopic studies of induced cartilage development and calcification**. *The Journal of Cell Biology* 1967, **35**(1):81-101.

22. Kirsch T, Wang W, Pfander D: **Functional differences between growth plate apoptotic bodies and matrix vesicles**. *Journal of Bone and Mineral Research* 2003, **18**(10):1872-1881.
